# Positive Regulation of TRAF6-Dependent Innate Immune Responses by Protein Phosphatase PP1-γ

**DOI:** 10.1371/journal.pone.0089284

**Published:** 2014-02-20

**Authors:** Amanda M. Opaluch, Monika Schneider, Chih-yuan Chiang, Quy T. Nguyen, Ana M. Maestre, Lubbertus C. F. Mulder, Ismael Secundino, Paul D. De Jesus, Renate König, Viviana Simon, Victor Nizet, Graham MacLeod, Susannah Varmuza, Ana Fernandez-Sesma, Sumit K. Chanda

**Affiliations:** 1 Infectious and Inflammatory Disease Center, Sanford-Burnham Medical Research Institute, La Jolla, California, United States of America; 2 Department of Microbiology and The Global Health and Emerging Pathogens Institute, Mount Sinai School of Medicine, New York, New York, United States of America; 3 Department of Pediatrics, University of California San Diego, La Jolla, California, United States of America; 4 Paul-Ehrlich-Institut, Federal Institute for Vaccines and Biomedicines, Research Group “Host-Pathogen Interactions”, Langen, Germany; 5 Skaggs School of Pharmacy and Pharmaceutical Sciences, University of California San Diego, La Jolla, California, United States of America; 6 Department of Cell and Systems Biology, University of Toronto, Toronto, Ontario, Canada; Virginia Tech University, United States of America

## Abstract

Innate immune sensors such as Toll-like receptors (TLRs) differentially utilize adaptor proteins and additional molecular mediators to ensure robust and precise immune responses to pathogen challenge. Through a gain-of-function genetic screen, we identified the gamma catalytic subunit of protein phosphatase 1 (PP1-γ) as a positive regulator of MyD88-dependent proinflammatory innate immune activation. PP1-γ physically interacts with the E3 ubiquitin ligase TRAF6, and enhances the activity of TRAF6 towards itself and substrates such as IKKγ, whereas enzymatically inactive PP1-γ represses these events. Importantly, these activities were found to be critical for cellular innate responses to pathogen challenge and microbial clearance in both mouse macrophages and human monocyte lines. These data indicate that PP1-γ phosphatase activity regulates overall TRAF6 E3 ubiquitin ligase function and promotes NF-κB-mediated innate signaling responses.

## Introduction

The sensing of foreign pathogens by pattern recognition receptors (PRRs) present on cells of the innate immune system serves as a first line of host defense against harmful microorganisms. Various PRRs are involved in this host immune response, including receptors belonging to the Toll-like receptor (TLR) family. Twelve mammalian TLRs have been characterized thus far, and their localization on the plasma membrane or on endolysosomal membranes affords each receptor access to pathogen-encoded ligands such as lipopolysaccharide (LPS; recognized by TLR4), flagellated proteins (recognized by TLR5), or virus- and bacteria-derived nucleic acids (recognized by TLR3, TLR7/8, and TLR9). Immune responses from endosomal TLRs, and in particular, TLR7, have been implicated in the control of RNA viruses including influenza virus, human immunodeficiency virus (HIV), and Sendai virus (SV) [1], [2]. Moreover, bacteria-derived nucleic acids, such as those from group A *Streptococcus* (GAS), have been shown to activate endosomal TLRs [2], [3].

Upon binding cognate ligands, TLR signaling is initiated via the interaction of cytoplasmic TIR (Toll/IL-1 receptor homology) domains with appropriate adaptor proteins including MyD88 (myeloid differentiation factor 88), TRIF/TICAM-1 (TIR containing adaptor molecule-1), TRAM (TRIF related adaptor molecule) and TIRAP/MAL (TIR domain containing adaptor protein) [4]. With the exception of TLR3, all TLRs, as well as IL-1R (interleukin-1 receptor), require an initial association with MyD88 in order to propagate downstream activation of proinflammatory cytokines and type I IFNs by NF-κB or IRF (interferon regulatory factor) transcription factors, respectively. Immediately following receptor ligation and association with MyD88, a downstream kinase cascade involving phosphorylation of IRAK (IL-1R associated kinase) proteins results in activation of the E3 ubiquitin ligase activity of TRAF6 (tumor necrosis factor receptor associated factor 6). Subsequently, TRAF6 catalyzes the K63-linked ubiquitination of substrates, including TRAF6 itself, IKKγ/NEMO (NF-κB essential modulator) and the MAP kinase, TAK1 (TGF-β-activated kinase 1) [5]–[8]. These upstream events are critical for activation of a multi-subunit complex referred to as the IKK signalosome, which is comprised of two kinases, IKKα and IKKβ, as well as the catalytically inactive IKKγ regulatory subunit [9]. Together, these IKK proteins coordinate the phosphorylation, ubiquitination, and degradation of inhibitory IκBα proteins, liberating NF-κB heterodimers to translocate into the nucleus and induce the transcription of pro-inflammatory target genes.

Within this inflammatory signaling pathway, TRAF6 has a critical role in integrating molecular information from multiple upstream receptors including IL-1R, CD40, TCR and TLRs, to induce downstream activation of NF-κB, AP-1 and IRF transcription factors [4], [10]–[12]. How TRAF6 is able to precisely interpret and process these signals to promote a robust innate immune response, while limiting inflammatory damage to host tissues, is still not completely defined. However, several enzyme complexes, protein interactions and post-translational modifications have been implicated in the regulation of this critical signaling event. A study by Deng and colleagues established that the ability of TRAF6 to conjugate K63-linked ubiquitin chains relies on an E2 complex containing two proteins: Ubc13 and Uev1A [5]. While both Ubc13 and Uev1A are critical for *in vitro* enzymatic activity of TRAF6, a conditional knockout of *Ubc13* in murine macrophages demonstrated that this protein is at least partially dispensable for TRAF6-mediated NF-κB signaling downstream of TLRs and IL-1R, implicating other molecular components in the regulation TRAF6 E3 ubiquitin ligase activity [13]. Similarly, a protein complex containing TAB1 and TAB2 is essential for the TRAF6-dependent ubiquitination of TAK1 [8], whereas the complement of factors regulating TRAF6-mediated ubiquitination of IKKγ is less well understood. Within this model, it is not clear if TRAF6 is differentially regulated via other post-translational modifications, and it is likely that additional molecules are involved in this process.

Here, we report the identification and characterization of protein phosphatase 1 (PP1) as a positive regulator of MyD88-dependent innate immune signaling and TRAF6 E3 ubiquitin ligase activity. PP1 is a type 1 serine threonine phosphatase previously shown to be important for multiple cellular processes including glycogen metabolism, mitosis, muscle contraction, as well as others [reviewed in [14] and [15]]. Each phosphatase holoenzyme is comprised of a catalytic subunit and a regulatory or inhibitory subunit, with the latter directing substrate specificity, subcellular localization, and enzymatic activity. The catalytic subunit of PP1 is expressed as α, β, γ1, and γ2 subunits, though the γ2 subunit is exclusively expressed in testis [16]. Recently, it was demonstrated that PP1-α and PP1-γ (PPP1CC) are important for the dephosphorylation of RIG-I and MDA5, which results in activation of these proteins [17]. PP1-α and PP1-γ were shown to interact with both RIG-I and MDA5, and the overexpression of these phosphatases resulted in increased production of IFNβ. In a separate study it was also reported that the α catalytic subunit interacts with the regulatory protein GADD34 to inhibit TNFR-induced NF-κB signaling by dephosphorylation of IKKα/β [18]; however, a functional role for PP1 subunits in TLR-mediated innate immune responses has not yet been described. Our results demonstrate a role for the γ catalytic subunit of PP1 (PP1-γ) in the positive regulation of MyD88-dependent NF-κB signaling events that augment proinflammatory immune activities. Specifically, our data reveal that the phosphatase PP1-γ enhances TRAF6 E3 ubiquitin ligase activity and is essential for the induction of effective innate responses to microbial infection.

### Ethics Statement

All procedures involving laboratory animals were approved by the Canadian Council on Animal Care.

## Materials and Methods

### Cell Lines and Tissue Culture

HEK293T cells were cultured in Dulbecco’s Modified Eagle Medium (DMEM) supplemented with 10% FBS, L-glutamine and penicillin/streptomycin. The HEK293T/TLR7/NF-κB luciferase reporter cell line was generated by transfecting HEK293T cells with an expression plasmid for TLR7, along with a 5X NF-κB luciferase reporter construct. HEK293T cells stably expressing hTLR4 or hTLR3 were obtained from Invivogen. THP-1 and RAW cells were cultured in RPMI-1640, supplemented with 10% FBS, L-glutamine and penicillin/streptomycin. RAW264.7 cells were transduced with LMP microRNA-adapted retroviral vectors (Thermo scientific) targeting PP1-γ, Unc93b1, MyD88, or GL2 to generate stable knockdown cell lines. BMDM were generated by culturing bone marrow cells in DMEM containing 10% FBS, L-glutamine, penicillin/streptomycin and 20% conditioned medium from L929 mouse fibroblasts for 7 days. For stimulations, R848, Flagellin, Poly I:C and LPS (Invivogen) and TNF-α, IL-1β (Cell Signaling) were used. MDDCs were grown in RPMI medium containing 10% FBS (HyClone; Thermo Scientific), 2 mM L-glutamine, 1 mM sodium pyruvate and 100 U/ml penicillin–100 µg/ml streptomycin (Gibco, Life Technologies) (complete DC medium) and supplemented with 500 U/ml human granulocyte-macrophage colony-stimulating factor (hGM-CSF) and 1,000 U/ml human interleukin 4 (hIL-4) (Peprotech).

### High-throughput cDNA Screening

A 384-well plate-based assay was optimized to identify cDNAs that have the capacity to positively regulate innate immune responses. For this assay, a focused library was generated. The library contained 1,179 cDNAs in total, and all cDNAs were obtained from the mammalian gene collection (MGC, http://mgc.nci.nih.gov/) and were in the mammalian expression vector pCMV-SPORT6. Eighty-six library cDNAs corresponded to genes that were not associated with PRR responses but that contained domains known to be required for innate immune signaling activation (e.g. TIR (toll IL-1 receptor), CARD (caspase activation and recruitment domain), LRR (leucine rich repeat), and others) [19]–[22]. Sixteen library cDNAs were selected based on their ability to reduce HIV replication or virus release in an HIV packaging screen (data not published). Lastly, 1,077 library cDNAs corresponded to genes whose cognate siRNAs enhanced HIV infectivity in a genome-wide HIV host factor screen completed by our lab [23]. This focused library was individually arrayed in 384-well plates such that each gene was assayed in duplicate for each cell line or luciferase reporter condition tested. Each plate also contained positive controls (p65, MAVS, IRF3-5D (constitutively active IRF3), IKK-SE (constitutively active IKK) or TRIF), negative controls (pcDNA3.1 vector, GFP, or pCherry vectors), and empty wells. The library was introduced into a HEK293T/NF-κB-luciferase cell line by high-throughput transfection with Fugene6 transfection reagent (Roche). Forty-eight hours post-transfection, Bright-Glo (Promega) was added in equal volumes to each well, and the luminescence associated with each sample was analyzed. The screen was run in duplicate, genes were selected for secondary confirmation assay by calculating the median of each plate, and a threshold of two standard deviations away from the median was used to designate ‘hits.’ The library was also counter-screened to identify cDNAs that influenced cell viability using ATPlite (Perkin Elmer). Genes associated with significant cytotoxicity were excluded from further studies.

### Plasmids and Vectors

Plasmids encoding p65, MAVS, pcDNA3.1 vector, GFP, and pCherry, were property of our lab and were all in CMV promoter-driven vectors. The pNiFty2 NF-κB luciferase reporter (Invivogen) construct was used for screening. For secondary assays of PP1-γ activity, an additional PP1-γ plasmid was obtained from Origene, and sequenced to determine integrity. This plasmid was further used for creation of the catalytically inactive mutant (PP1-γ D64N), using a site-directed mutatgenesis kit (Stratagene). 3X-FLAG tagged TLR constructs were generated by PCR amplification of MyD88, TRAF6, TRAF3, TBK1, TANK, IKKγ and IKKε genes, and ligation into the pEGFP-N1 vector, where GFP had been replaced with 3X FLAG (N-terminal). TRAF6 truncation mutants were generated by PCR amplification of the relevant fragments with the addition of an in-frame N-terminal FLAG sequence. Each fragment was cloned into the pcDNA3.1(+) mammalian expression vector (Life Technologies) using EcoRI and NotI sites.

### RNA Interference

Double-stranded RNA duplexes targeting human MyD88, p65 and PP1-γ were purchased from Qiagen. The TNFR siRNA was a SMARTpool purchased from Dharmacon. Negative control siRNAs used were either from Qiagen or were previously described [24]. HEK293 cells were transfected using Lipofectamine 2000 according to manufacturer’s protocols, and THP-1 cells were transfected using HiPerfect according to manufacturer’s protocols. Cells were assayed for gene knockdown either 48 or 72 hours post-transfection, depending on experiment or assay completed.

### Enzyme-linked Immunosorbent Assay (ELISA)

Three days post-transfection of HEK293T/TLR7/NF-κB with siRNAs, and after stimulation with R848 for 12 hours, a human IL-8 immunoassay (eBioscience) was performed according to the manufacturer’s instructions. RAW264.7 stable knockdown cell lines were stimulated with LPS for 16 hours and a murine IL-6 immunoassay (R&D Systems, Inc.) was performed according to the manufacturer’s instructions.

### Luciferase Reporter Assays

The HEK293T/TLR7/NF-κB luciferase reporter cell line was reverse transfected with siRNA or cDNA using Lipofectamine 2000 (Life Technologies) and analyzed in triplicate for each assay. Forty-eight hours post transfection, the cells were stimulated (siRNA) or left unstimulated (cDNA), and the luciferase reporter activity was quantified with Britelite Plus (PerkinElmer) sixteen hours post-stimulation. The same transfection conditions were used for the cytotoxic assay. Three days post transfection, viability of the cells was quantified with ATP Lite (PerkinElmer). Both the luciferase assay and cytotoxic assay were quantified by using the PHERAstar luminometer (BMG Labtech).

### Realtime PCR

Total RNA was extracted from cells using RNeasy Mini or RNeasy 96 Kit according to the manufacturer’s instructions (Qiagen). RNA samples were reverse transcribed using the QuantiTect Reverse Transcription Kit (Qiagen). PCR products were detected using the Power SYBR® Green PCR Master Mix (Applied Biosystems) and an ABI 7900HT. Relative mRNA abundances were calculated by the Δ*C_T_* method using the housekeeping gene TATABP or rps11 to normalize the results. The results were plotted as mean relative expression. The primers used were - TATBP: 5′-CCACTCACAGACTCTCACAAC-3′, 5′-CTGCGGTACAATCCCAGAACT-3′; ICAM-1: 5′-TGGCCCTCCATAGACATGTGT-3′, 5′-TGGCATCCGTCAGGAAGTG-3′ TNF-α: 5′-ATGAGCACTGAAAGCATGATCC-3′, 5′-GAGGGCTCATTAGAGAGAGGTC-3′; IκBα: 5′-CCCAAGCACCCGGATACAG-3′, 5′GTGAACTCCGTGAACTCTGAC-3′; PP1-γ: 5′-CTCAACATCGACAGCATTATCCA-3′, 5′-CGAGACTTTAAGCACAGTCCTC-3′; IL-8∶5′-TTTTGCCAAGGAGTGCTAAAGA-3′, 5′-AACCCTCTGCACCCAGTTTTC-3′; rps11∶5′-GCCGAGACTATCTGCACTAC-3′, 5′-ATGTCCAGCCTCAGAACTTC-3′; RANTES: 5′-TTGCCAGGGCTCTGTGACCA-3′, 5′-AAGCTCCTGTGAGGGGTTGA-3′; IP-10∶5′-TCCCATCACTTCCCTACATG-3′, 5′-TGAAGCAGGGTCAGAACATC-3′; murine PP1-γ: 5′-TGTCATGGAGGTTTATCACCAGA-3′, 5′-CGGGGTCAGACCACAAAAGA-3′; murine β-actin: 5′- ACGGCCAGGTCATCACTATTG-3′, 5′-CAAGAAGGAAGGCTGGAAAAGAG-3′.

### p65 Nuclear Translocation Assay

HEK293T/TLR7 cells were plated in chambered coverglass systems (Lab-Tek cat#155411) and were reverse transfected with siRNAs. Two days post-transfection, cells were stimulated with R848 at a concentration of 0.5 µM for 40 minutes. The cells were then washed with PBS, fixed with 3.7% paraformaldehyde, and permeabilized with a solution of 0.1% Saponin in PBS and blocked with a solution of 0.1% Saponin and 2.5% Normal Goat Serum in PBS. The latter solution was used for all subsequent washes and for antibody incubations. Cells were incubated with primary antibody against p65/RELA (Santa Cruz, sc-8008) for 1 hour and then secondary antibody (goat anti-mouse Alexa 488; Life Technologies, A11029) for 2 hours, both at room temperature. The cells were washed with blocking solution and PBS, then overlaid with VECTASHIELD with DAPI (Vector Laboratories, H-1200). The samples were imaged using an inverted TE300 Nikon wide field fluorescence microscope.

### Western Blotting, Immunoprecipitation, and *in vitro* Ubiquitination Assays

For western blotting, cells were seeded on various-sized plates, and stimulated as indicated. Cells were harvested in lysis buffer (50 mM HEPES, pH 7.4, 100 mM NaCl, 1% Triton-X 100, 50 mM NaF, 5 mM sodium orthovanadate, 1 mM PMSF, 5 mM EDTA, STI/AL) and whole cell lysates (WCLs) were collected by centrifugation. For westerns and co-IPs, anti-FLAG (Sigma), anti-PP1-γ, anti-TAK1, anti-IKKγ/NEMO, anti-TRAF6 anti-IKKβ and all normal IgGs (all Santa Cruz) were used. Additionally, anti-IκBα, anti-phospho-TAK1 (Thr184/187), anti-Ubiquitin (PD41), anti-p38, anti-phospho-p38 (Thr180/Tyr182), anti-phospho-IKKβ (sc-7607), anti-p53 and anti-actin (all Cell Signaling) were utilized. The secondary antibodies used were HRP-conjugated goat anti-rabbit (Bio-Rad), goat anti-mouse (Bio-Rad), bovine anti-goat (Jackson ImmunoResearch) and light-chain specific rabbit (Jackson ImmunoResearch). For co-immunoprecipitation (co-IP), cells were harvested using co-IP lysis buffer (50 mM Tris-HCl, pH 7.4, 250 mM NaCl, 1 mM EDTA, 1% Triton-X 100) supplemented with complete protease inhibitor and phosphatase inhibitor. 3XFLAG-tagged proteins were immunoprecipitated using anti-FLAG M2 agarose beads (Sigma), and endogenous proteins were immunoprecipitated using ProteinG Sepharose (Sigma) and the indicated antibodies. WCLs were immunoprecipitated overnight at 4°C with rotation, or for 2 hours at 4°C with rotation for *in vitro* ubiquitination assays. All WCLs were resolved on NuPAGE Tris-Glycine gels, transferred to a PVDF membrane using a semi-dry transfer apparatus, probed overnight at 4°C with primary antibodies, then incubated with secondary antibody for 2 hours at room temperature. *In vivo* ubiquitin assays were run by overexpressing FLAG-tagged TRAF6 or IKKγ constructs with HA-tagged ubiquitin and with or without overexpressed PP1-γ. Twenty four hours following transfection, the cells were lysed as described above, and an aliquot was collected for the WCL. A 1% SDS buffer was added to the remaining lysate, to a final concentration of 0.75% SDS, and the samples were boiled for 15 minutes to eliminate any noncovalent interactions. The lysates were then diluted with a buffer that did not contain detergent, and the immunoprecipitation was carried out as described above. *In vitro* ubiquitination assays were completed as previously described [25].

### Lentiviral Vector Construction and Virus Production

For induced expression of PP1-γ in primary human monocyte derived dendritic cells (MDDCs), cDNAs encoding PP1-γ WT and PP1-γ D64N where cloned in the lentiviral vector pVIN4Δrep using standard molecular biology techniques. Viruses were produced by transfecting 293T cells with the PP1-γ lentiviral vectors alongside plasmids for HIV-1 gag-pol (psPAX or pNL4-3 gag-pol) and a plasmid encoding VSV-G envelope [26]. Supernatants were harvested 48 and 72 hours after transfection, 0.45 µM filtered, and concentrated by centrifugation through a 20% sucrose cushion at 15000 g for 5–6 hours. Viruses were titrated using EnzChek Reverse Transcriptase Assay Kit (Molecular Probes).

### Human Monocyte Derived Dendritic Cells

Peripheral blood mononuclear cells (PBMC) were isolated by Ficoll density gradient centrifugation (Histopaque; Sigma Aldrich) from buffy coats of healthy human donors (Mount Sinai Blood Donor Center and New York Blood Center). CD14^+^ cells were purified using anti-human CD14 antibody-labeled magnetic beads and MiniMACS liquid separation columns (Miltenyi Biotech). After elution, CD14^+^ cells were incubated at 37°C for 5 days at a concentration of 10^6^ cells/ml, and a pool of monocyte-derived dendritic cells (MDDCs) was generated. MDDCs were transduced with pVIN4Δrep lentiviral vectors by spinoculation (1300 rpm, 3 h). Twenty-four hours later the expression of the transduced proteins was induced by doxycycline (0.5 µg/ml).

### Macrophage Total Killing Assay

RAW264.7 murine macrophage cell lines with stable silencing of PP1-γ, MyD88, Unc93B1, or GL2 (negative controls, NCTLs) were seeded at 5×10^5^ cells per well in 24 well plates the day prior to the assay. One hour before adding bacteria, cells were washed twice with PBS and 0.5 ml of RPMI +2% FBS was added to each well. GAS serotype M49 strain NZ131 [27] was pre-opsonized with 80% human plasma for 45 min at 37°C and added to cells at multiplicity of infection (MOI) of 1 bacteria per macrophage and a final concentration of 2% human plasma. Plates were then centrifuged at 2,000 rpm to ensure bacterial contact with the macrophages. Plates were incubated for 4 h at 37°C in 5% CO_2_. Cells were lysed with 0.025% Triton-X 100 in PBS and serial dilutions were plated on agar for enumeration of surviving bacterial colony forming units (cfu).

### Animals

Mice were bred using standard animal husbandry. The *Ppp1cc* mutant allele has been propagated in a CD-1 background (Charles River Laboratories). Mutant and wild type mice were identified by PCR genotyping as described [28]; alternatively, tail biopsies were boiled for 30 minutes in 100 ml of 50 mM NaOH, neutralized with 30 ml of 1M Tris buffer, pH 6.8, and analyzed by PCR with primers Int4 (5′-ctcaggccaatgctgtctgc-3′) (common forward primer), Neo-3R (5′-agcctctgagcccagaaabc-3′) (mutant allele reverse primer) and D486 (5′-actcatagccatcttcaaccacc-3′) (wild type allele reverse primer). Adult males between 3 and 9 months of age were used for all experiments.

## Results

### Identification of PP1-γ as a Critical Factor for MyD88-dependent Innate Immune Responses

**Figure 1:**
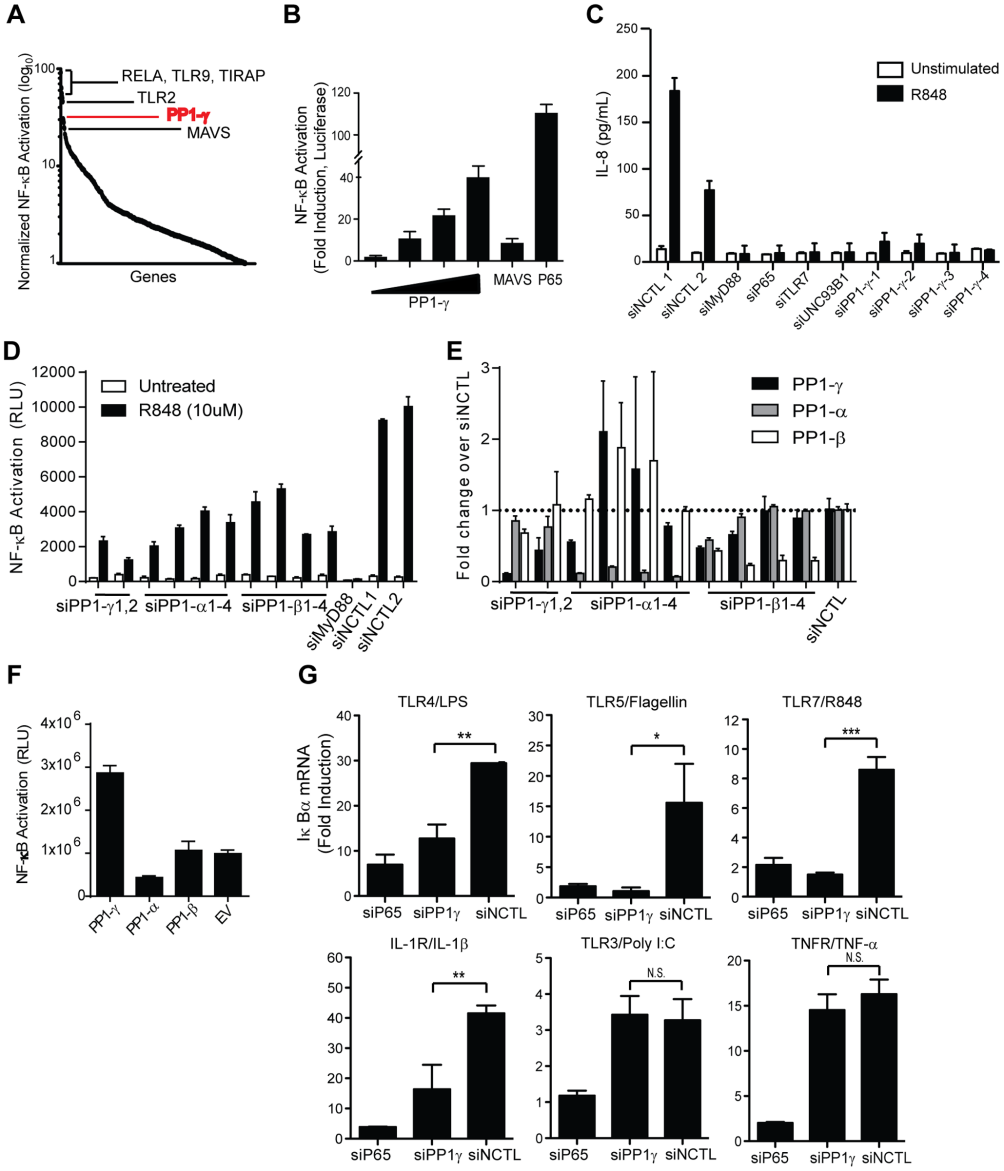
A role for PP1-γ in MyD88-dependent Toll/IL-1R activation. A) A library of approximately 1,200 cDNAs (*Genes*, x-axis) was arrayed in 384-well plates and individually transfected into HEK293T cells stably expressing an NF-κB-luciferase reporter (HEK293T/NF-κB-luc) and the ability of each cDNA to ectopically activate the NF-κB reporter was measured. Also see Supplemental Experimental Procedures. B) HEK293T cells were transfected with vector, MAVS, or p65 (60 ng/well), and PP1-κ was transfected in increasing amounts (10, 20, 40, 60 ng/well, respectively). Forty-eight hours post-transfection, luciferase values were evaluated. Fold NF-κB activation was calculated relative to vector-transfected samples. C) HEK293T/NF-κB-luc cells stably expressing TLR7 were transfected with indicated siRNAs, stimulated with R848 (3 µM) for 12 hours, and secreted IL-8 was quantified by ELISA. Also see Figure S1B. D) HEK293T/NF-κB-luc cells stably expressing TLR7 were transfected with indicated siRNAs, stimulated with R848 (10 µM) for 16 hours. Luciferase values were then measured. E) HEK293T/NF-κB-luc cells stably expressing TLR7 were transfected with siRNAs (indicated on the x-axis) for 48 hours and the expression of each PP1 subunit was measured by qPCR. Values shown as the fold-change over cells transfected with a negative control. F) HEK293T cells were transfected with NF-κB-luc and vectors containing PP1-γ, PP1-α or PP1-β for 24 hours, then luciferase values were measured. G) HEK293T/NF-κB cells stably expressing TLR3, TLR4, or TLR7 were reverse transfected with the indicated siRNAs. Forty-eight hours post-transfection, cells were stimulated for 3 h with LPS (TLR4, 100 ng/mL), Flagellin (TLR5, 100 ng/mL), R848 (TLR7, 10 µM), IL-1β (IL-1R, 10 ng/mL) or TNF-α (TNFR, 10 ng/mL), or for 6 h with poly I:C (TLR3; 50 µg/mL). For evaluation of TLR5, IL-1R, and TNFR stimulation, HEK293T/NF-κB cells stably expressing TLR7 were used. Relative levels of IκBα mRNA were evaluated by RT-PCR. Also see Figure S1C. Data from A–E are representative of at least three independent experiments; by two-tailed student’s t test, *P*≤0.05 = *, *P*≤0.01 = **, *P*≤0.001 = ***, N.S. = not significant. Bar graphs in (B), (C), and (D) are presented as mean ± SD. Bar graphs in (E) are presented as mean relative mRNA levels ± SD.

In an effort to identify innate regulatory molecules, we assembled a gain-of-function sub-genomic library containing genes that were likely to be regulators of immune signaling. Specifically, cDNAs were selected for genes that demonstrated significant activities in high-throughput viral restriction screens [24], or that contained domains associated with innate signaling [21]. This customized library was comprised of 1,200 genes under the control of a CMV enhancer/promoter. Library cDNAs were individually arrayed and screened in a cell-based assay to test for the ability of each gene to activate an NF-κB luciferase reporter (Figure 1A, Supplementary Table 1). Using this methodology, we confirmed the activities of several known innate signaling genes (e.g. TLR9, TLR2, TIRAP) and also identified a number of putative innate regulatory genes. A subset of these putative innate factors were subsequently tested in several reporter-based confirmation assays; consistently, we found the gamma catalytic subunit of protein phosphatase 1 (PP1-γ) was a top hit in these assays, and thus, was likely a potent activator of proinflammatory signaling. For example, ectopic expression of PP1-γ in HEK293T cells harboring an NF-κB luciferase reporter construct (HEK293T/NF-κB-luc) resulted in the induction of NF-κB luciferase reporter activity in a dose-dependent manner, up to 40-fold over control (Figure 1B). In this instance, expression of PP1-γ activated the reporter to a greater extent than expression of mitochondrial antiviral signaling (MAVS) protein [29], but not as highly as the ectopic expression of p65.

To evaluate the function of endogenous PP1-γ in proinflammatory signaling downstream of TLR activation, we transfected HEK293T/NF-κB-luc cells stably expressing TLR7 (HEK293T/TLR7/NF-κB-luc) with siRNAs targeting PP1-γ and stimulated cells with the synthetic TLR7 ligand, R848. In this system, we verified depletion of PP1-γ mRNA following RNAi, and observed that PP1-γ silencing attenuated R848-induced activation of the NF-κB-luciferase reporter, as well as R848-induced IL-8 mRNA upregulation (Supplementary Fig. S1A). Furthermore, PP1-γ RNAi inhibited TLR7-mediated IL-8 cytokine secretion (Figure 1C and Supplementary Fig. S1B).

Next, to determine whether this regulatory role was specific to the γ subunit, PP1-α and PP1-β siRNAs were transfected into HEK293T/TLR7/NF-κB-luc cells, and NF-κB activity was measured after R848 stimulation. Similar to the decreased NF-κB activation observed with PP1-γ silencing (Figure 1C), knockdown of PP1-α or PP1-β also resulted in attenuated NF-κB activation (Figure 1D). This effect was not due to nonspecific targeting of the siRNAs (Figure 1E). However, in contrast to PP1-γ, overexpression of PP1-α or PP1-β did not result in NF-κB activation (Figure 1F). These results indicated that multiple PP1 subunits may play a role in the regulation of NF-κB signaling, but that PP1-γ was the only subunit that was necessary and sufficient for activation of the pathway. Together, these results suggested that PP1-γ was a positive regulator of TLR-mediated proinflammatory responses. However, it was unclear whether PP1-γ was uniquely necessary for TLR signaling, or whether this phosphatase was more generally required for NF-κB activation.

A common feature of most innate signaling pathways is their capacity to initiate downstream transcription of NF-κB-dependent target genes to initiate proinflammatory responses, but the required receptor-proximal components are more diverse. For instance, TLR3 exclusively initiates signaling via the TRIF adaptor protein, TNFR signals via the TRADD and RIP adaptor proteins, and TLRs 4, 5, and 7 initiate signaling via MyD88 [4], [30], [31]. To determine the range of proinflammatory signaling pathways affected by PP1-γ activity, we evaluated the effects of PP1-γ RNAi on NF-κB-dependent target gene induction downstream of multiple TLRs, IL-1R, and TNFR by stimulating HEK293T TLR cell lines with poly I:C (TLR3), LPS (TLR4), flagellin (TLR5), R848 (TLR7), IL-1β (IL-1R), and TNF-α (TNFR). Silencing of PP1-γ significantly attenuated the early induction of IκBα mRNA downstream of TLR4/5/7 and IL-1R, but TLR3- and TNFR-mediated responses remained intact (Figure 1G). Furthermore, induction of TNF-α downstream of TLR4/5/7 was impaired by PP1-γ silencing, while TLR3-mediated TNF-α mRNA induction was not significantly changed (Supplementary Fig. S1C). Since PP1-γ is dispensable for MyD88-independent signaling downstream of TLR3 and TNFR, these data indicate that PP1-γ is exclusively required for TLR4/5/7- and IL-1R-mediated proinflammatory signaling, and thus is likely a critical regulator of innate responses governed by MyD88.

### Catalytic Activity of PP1-γ is Essential for TLR-dependent NF-κB Activation

**Figure 2:**
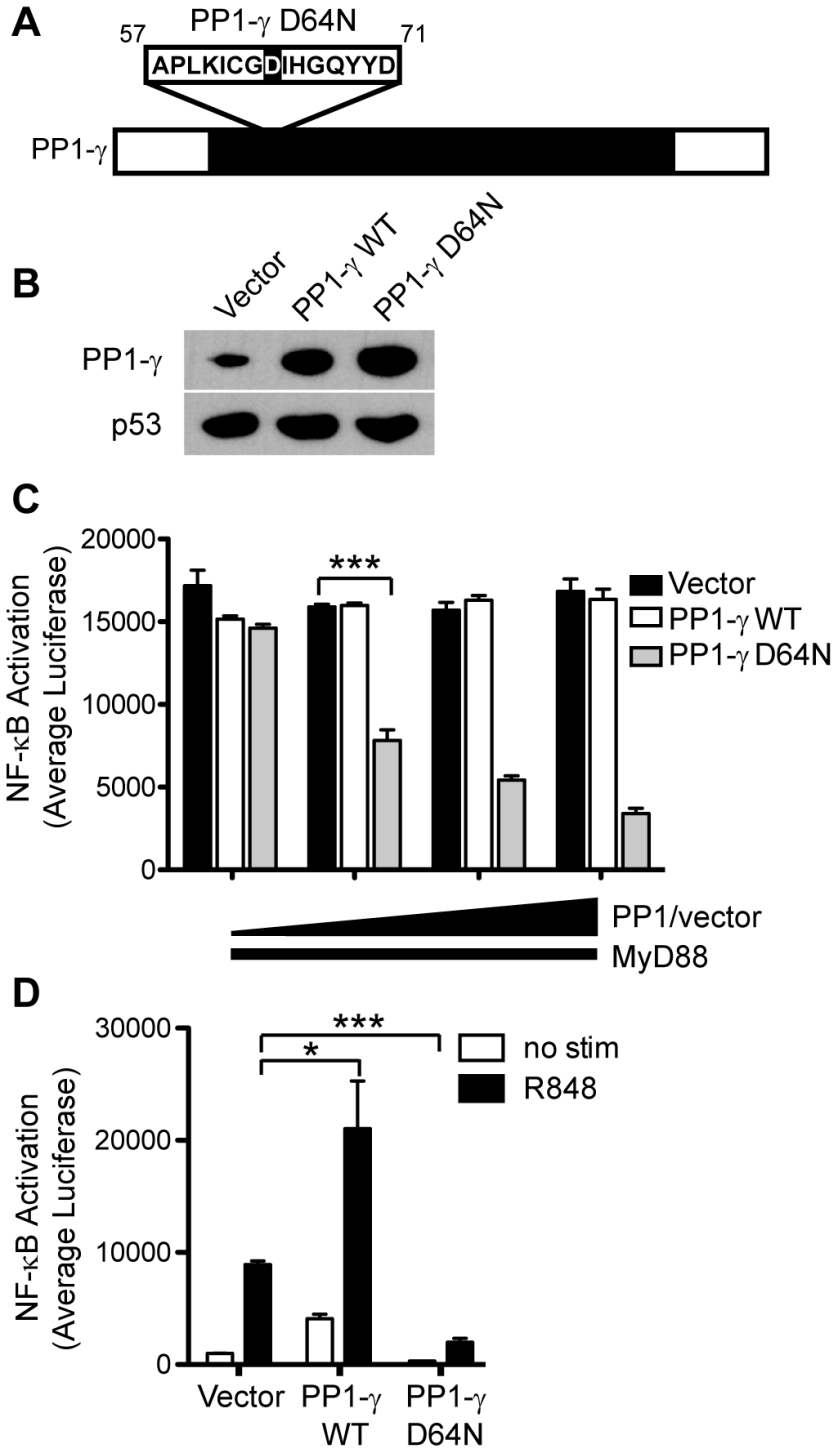
Catalytic activity of PP1-γ is necessary for TLR-mediated inflammatory responses. A) A schematic diagram of the PP1-γ gene. Non-conserved N- and C-terminal regions of PP1-γ are defined in white, while the conserved catalytic core is depicted in black. A region of the catalytic core is enlarged to show mutation of residue 64 from aspartic acid (D) to asparagine (N), described as “PP1-γ D64N.” Wild type PP1-γ is described as “PP1-γ WT.” B) HEK293 cells were transfected with plasmids encoding vector control, PP1-γ WT, or PP1-γ D64N. Forty-eight hours post-transfection, cells were harvested for immunoblotting with antibodies against PP1-γ or p53. C) HEK293T/NF-κB-luc cells were transfected with a constant concentration of MyD88 (40 ng/well), and increasing concentrations of vector control, PP1-γ WT, or PP1-γ D64N (0, 5, 10, 20 ng/well). Average luciferase values were evaluated 48 hours post-transfection. D) HEK293T/TLR7/NF-κB-luc cells were transfected with vector control, PP1-γ WT, or PP1-γ D64N as in (C). Forty-eight hours post-transfection, cells were stimulated for 16 h with R848 (10 µM), and average luciferase values were measured. Data from (B–D) are representative of at least three independent experiments; by two-tailed student’s t test, *P*≤0.05 = *, *P*≤0.001 = ***. Bar graphs in (C) and (D) are presented as the mean ± SD.

PP1 isoforms have a high degree of sequence similarity within the catalytic core that contribute to the metal-dependent dephosphorylation of PP1 substrates; however, the N- and C-termini of each protein contain subunit-specific divergent sequences [[32] and reviewed in [15]]. Of the residues within the catalytic core that are critical for enzymatic activity, several invariant aspartic acid and histidine residues are shared not only between the three PP1 catalytic subunits, but also conserved between other eukaryotic serine threonine phosphatases, bacteriophage phosphatases and *E. coli* adenosinetetraphosphatase [33]. Among these residues, mutation of aspartic acid (D) at position 64 to asparagine (N) results in an ∼10^3^-fold loss in catalytic activity of PP1 as measured using phosphorylase *a* as a substrate (Figure 2A) [32].

To determine whether the phosphatase activity of PP1-γ was necessary for MyD88-dependent proinflammatory signaling, we tested the effect of the catalytically dead PP1-γ D64N mutant (PP1-γ D64N) on NF-κB responses. When transiently expressed, protein levels of PP1-γ D64N were comparable to that of the wild-type catalytically active PP1-γ (PP1-γ WT) (Figure 2B). Co-transfection of PP1-γ WT and a plasmid encoding MyD88 caused robust activation of the NF-κB reporter, but this response was strongly inhibited by transfection of increasing doses of PP1-γ D64N (Figure 2C). These data suggested that the catalytically inactive PP1-γ D64N mutant acted as a dominant negative for MyD88-induced TLR signaling. To further test the repressive capacity of mutant PP1-γ, HEK293T/TLR7/NF-κB-luc cells were transfected with PP1-γ WT or PP1-γ D64N, and NF-κB responses were evaluated following R848 stimulation (Figure 2D). Consistent with previous data, ectopic expression of catalytically active PP1-γ augmented TLR7-induced activation of NF-κB, while PP1-γ D64N strongly suppressed these transcriptional responses, indicating that loss of PP1-γ enzymatic activity critically impairs TLR7-dependent proinflammatory signaling events.

### Biochemical Mapping of PP1-γ Activity upon TLR-dependent NF-κB Signaling

**Figure 3:**
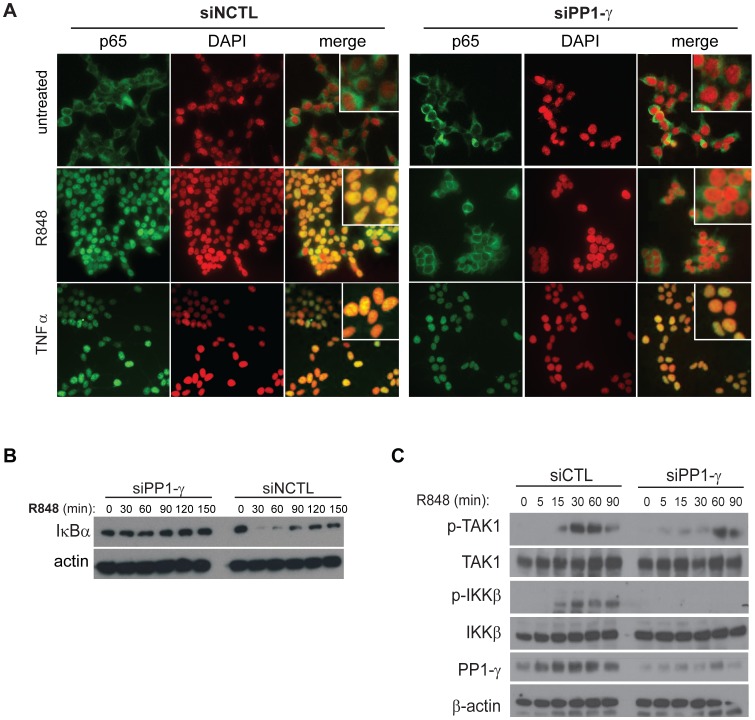
PP1-γ silencing impairs NF-κB and MAPK signaling events downstream of TLR activation. A) HEK293T/TLR7/NF-κB-luc cells were reverse transfected with the indicated siRNAs. Forty-eight hours post-transfection, cells were left untreated, or stimulated with R848 (0.5 µM) or TNF-α (10 ng/mL) for 40 minutes, then fixed and stained. Nuclear translocation of p65 was evaluated by immunofluorescence (nuclei (red) = DAPI; p65 (green)). B–C) HEK293T/TLR7/NF-κB-luc cells were reverse transfected with the indicated siRNAs. Forty-eight hours later, cells were stimulated with R848 (10 µM) for the indicated time points, and whole cell lysates were collected and used for immunoblotting with the indicated antibodies. Data shown are representative of at least three independent experiments.

Our results demonstrated that PP1-γ was a critical regulator of proinflammatory signaling mediated by multiple TLRs. In an effort to functionally map the role of PP1-γ in a MyD88-dependent signaling pathway, we elected to evaluate the effects of PP1-γ RNAi downstream of TLR7 because this receptor is highly expressed in immune-sensing cell types and is vital for antiviral host defense [34]. Initially, we measured the effect of PP1-γ silencing upon R848-induced p65 nuclear translocation and IκBα degradation. Typically, TLR-mediated nuclear accumulation of p65 occurs with 40 minutes of stimulation, but PP1-γ RNAi abrogated this response (Figure 3A). Similarly, the degradation and re-synthesis of IκBα following TLR7 stimulation was completely prevented by PP1-γ silencing (Figure 3B). In contrast, when cells were stimulated through TNFR, RNAi against PP1-γ had no effect on TNF-α-induced p65 nuclear translocation or IκBα degradation kinetics (Figure 3A and Supplementary Fig. S2). This data was consistent with our observation that PP1-γ selectively regulates MyD88-dependent signaling.

Next, we evaluated the effects of PP1-γ silencing on the phosphorylation-dependent activation of a number of essential kinases in the canonical TLR signaling pathway, including IKKβ and TAK1. We observed a significant reduction of TLR7-dependent phosphorylation of IKKβ when PP1-γ expression was knocked down (Figure 3C), and furthermore, ligand-induced phosphorylation of TAK1 at residues Thr184/187 in the kinase activation loop was significantly altered by siRNAs targeting PP1-γ (Figure 3C). TAK1 is a MAP kinase (MAPK) that is required for the MyD88-mediated activation of the IKK signalosome, as well as an ubiquitin-dependent kinase upstream of MAPK p38 [8]. These results imply that PP1-γ is functionally required for the TLR-mediated activation of TAK1, which is regulated by TAB1/2/3 in complex with TRAF6 [8], [35]. Interestingly, our ligand profiling analysis (Figure 1G and Supplementary Fig. S1C) indicated that PP1-γ was critical for proinflammatory signaling initiated via multiple MyD88-dependent receptors that all rely on TRAF6 to transmit downstream signals. Together, our genetic and biochemical data both support the restricted activity of PP1-γ in signaling pathways that exclusively utilize the MyD88 signaling adaptor, and suggest that this phosphatase acts upon or upstream of the TRAF6 E3 ubiquitin ligase complex.

### Biochemical Characterization of the Interaction between PP1-γ and TRAF6

**Figure 4:**
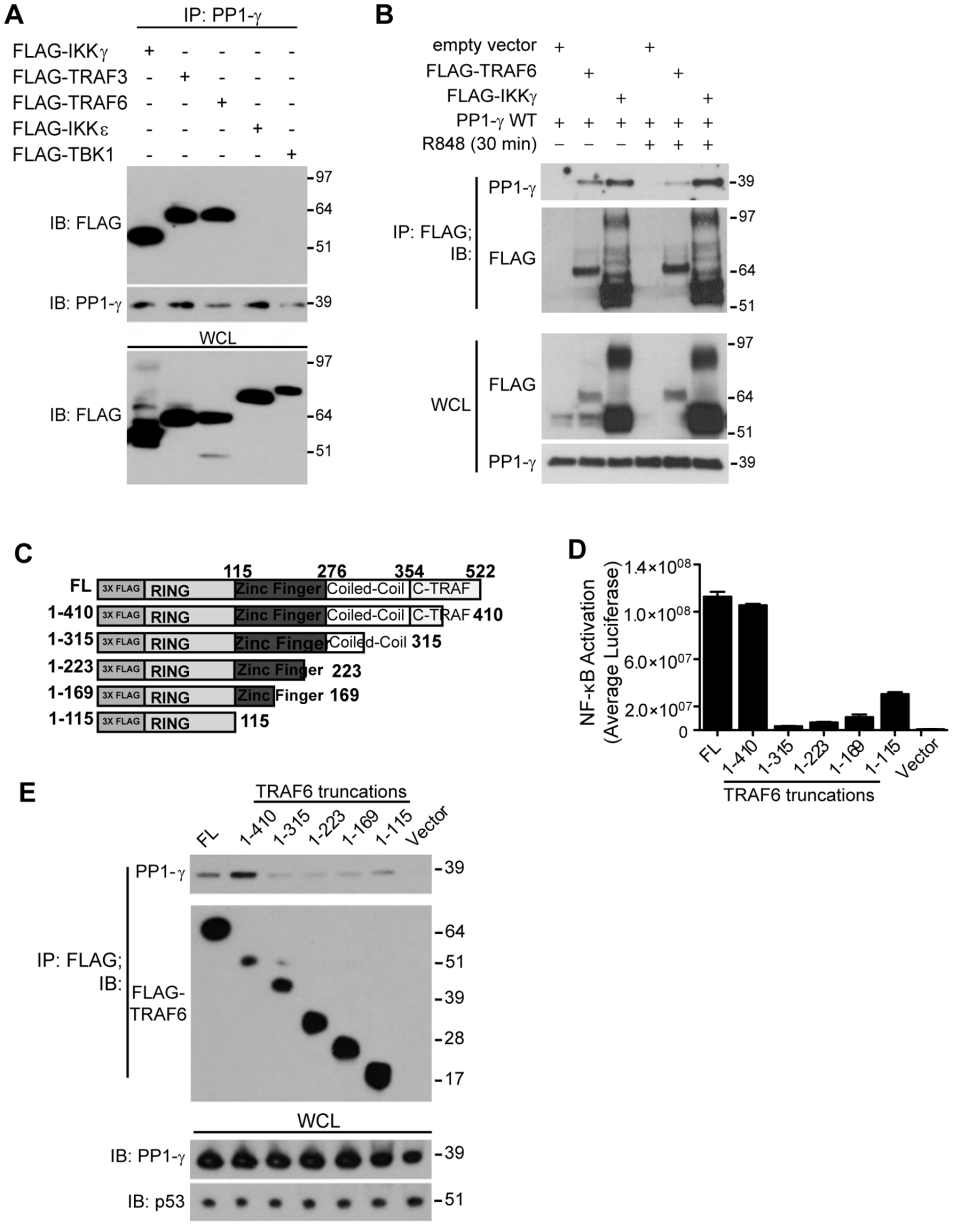
PP1-γ physically associates with TLR pathway members and TRAF6. A) HEK293T cells were transfected with 3X-FLAG tagged plasmids encoding IKKγ, TRAF3, TRAF6, IKKε, and TBK1, and whole cell lysates were harvested for immunoprecipitation (IP) of endogenous PP1-γ. Immunoprecipitates were subjected to SDS-PAGE and immunoblotting with anti-FLAG or anti-PP1-γ antibodies. B) HEK293T/TLR7 cells were transfected with an empty vector, FLAG-IKKγ or TRAF6 and PP1-γ WT or PP1-γ DN, then stimulated with R848 (10 µM) for 30 min. The cells were lysed and immunoprecipitated for FLAG, then immunoblotted for PP1-γ. C) Schematic diagram of wild-type TRAF6 and truncation mutants. D) HEK293T/NF-κB-luc cells were transfected with 100 ng of full length (FL) TRAF6, truncation mutants, or vector control. Forty-eight hours post-transfection, average luciferase values were evaluated. E) HEK293T cells were transfected with full length (FL) TRAF6 or truncation mutants together with 1 ug of PP1-γ WT. Lysates were harvested and used for FLAG immunoprecipitation followed by SDS-PAGE and immunoblotting with a PP1-γ antibody. Data in (A–B, D–E) are representative of at least three independent experiments.

To determine if PP1-γ regulates TLR-mediated NF-κB signaling through TRAF6, we evaluated a potential physical interaction of PP1-γ with multiple known TLR pathway components associated with the TRAF6 complex. Initially, protein associations were studied by co-immunoprecipitation of endogenous PP1-γ and ectopically-expressed 3XFLAG-tagged constructs (Figure 4A). By immunoprecipitation, we were able to demonstrate a physical association between PP1-γ and IKKγ (NEMO), TRAF3, and TRAF6, but were unable to detect an interaction with either IKKε or TBK1 (Figure 4A). Importantly, we were able to confirm an endogenous interaction between PP1-γ and IKKγ or TRAF6, but not between PP1-γ and TRAF3 (unpublished observations). We further investigated whether TLR7 stimulation disrupted the observed interaction between PP1-γ and TRAF6 or IKKγ, and we found that after stimulation with R848, PP1-γ remained in association with these proteins (Figure 4B). The physical association of PP1-γ with TRAF proteins and the TRAF6 substrate IKKγ further supports a potential role for this phosphatase as a regulator of TRAF6-dependent NF-κB-associated immune responses.

Structurally, TRAF6 is comprised of an amino-terminal RING finger domain, followed by a region containing four zinc finger motifs, a coiled-coil domain (or TRAF-N domain), and a highly conserved carboxyl-terminal TRAF-C domain. Together with the first zinc finger, the RING domain is responsible for the E3 ubiquitin ligase activity of TRAF6, whereas the TRAF-C domain controls protein oligomerization and binding to upstream TRAF6-interacting proteins. Furthermore, it has been shown that the E2 conjugating enzyme Ubc13 can interact with both the RING domain and the first zinc finger of TRAF6, as well as regions of the coiled-coil domain, suggesting a bipartite binding motif that facilitates TRAF6 E3 activity [36], [37]. Interestingly, an additional study has implicated an inhibitory TRAF6 intramolecular interaction involving the RING/zinc finger domains and the coiled-coil domain that potentially retains TRAF6 in a “closed” and inactive conformation [38].

To map the region of TRAF6 that is responsible for the interaction with PP1-γ, we generated a series of truncation mutants based on the domain structure of TRAF6, and cloned each mutant into a mammalian expression vector containing an amino terminal FLAG tag (Figure 4C). When these constructs were expressed in HEK293T/NF-κB-luc cells, full length TRAF6, as well as a mutant containing a partial TRAF-C domain (TRAF6 1–410), was competent to activate NF-κB, an observation that was consistent with previous reports (Figure 4D). However, the remaining mutants containing partial or full truncations of the coiled-coil or zinc finger domains (TRAF6 1–315, 1–223, 1–169, 1–115) displayed significantly impaired abilities to activate pro-inflammatory signaling. Importantly, when cells were co-transfected with TRAF6 truncation mutants and utilized for FLAG immunoprecipitation, an interaction with PP1-γ was observed for full length TRAF6 and the partial TRAF-C domain mutant (TRAF6 1–410); this interaction was considerably and consistently reduced when all other TRAF6 mutants (TRAF6 1–315, 1–223, 1–169, 1–115) were co-expressed (Figure 4E). This data implies that PP1-γ may interact with TRAF6 via residues 315–354 of the coiled-coil domain, or residues 354–410 of the TRAF-C domain, and it suggests that one of the potential consequences of this interaction is TRAF6 oligomerization, and the conformational change may enhance its ubiquitin ligase activity and promote activation of NF-κB signaling.

### PP1-γ Positively Regulates the E3 Ubiquitin Ligase Activity of TRAF6

**Figure 5:**
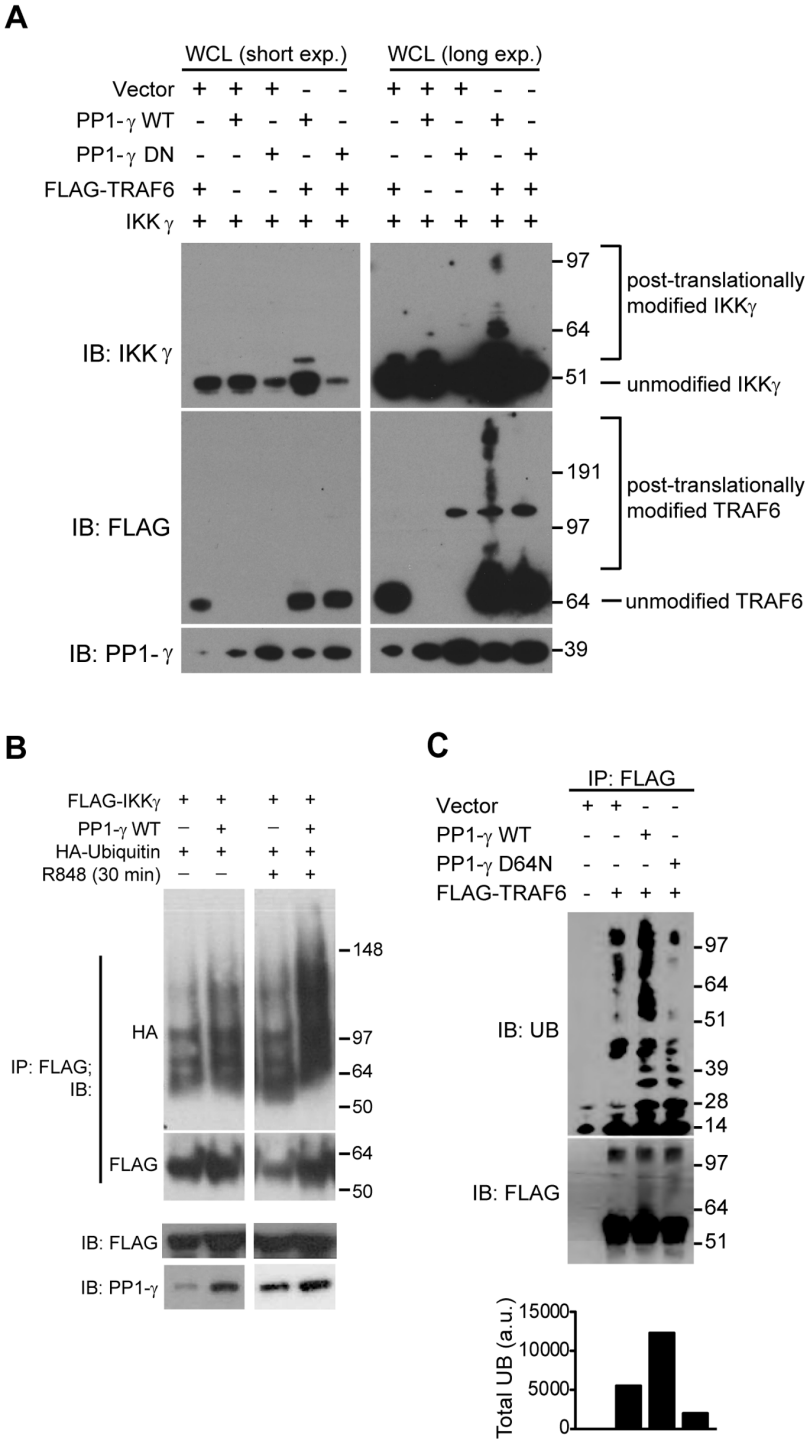
PP1-γ augments TRAF6 ubiquitin ligase activity. A) HEK293T cells were transfected with different combinations of the indicated plasmids as shown, and for each immunoblot, there is a short exposure (*short exp*.) and a long exposure (*long exp*.) for the purpose of observing higher molecular weight IKKγ and TRAF6 bands indicative of post-translational modifications such as phosphorylation or ubiquitination. IKKγ and TRAF6 immunoblots are labeled as “unmodified” or “post-translationally modified” to indicate different species. B) HEK293T cells were transfected with indicated plasmids and whole cell lysates were harvested for immunoprecipitation (IP) of FLAG-IKKγ. Immunoprecipitates were subjected to SDS-PAGE and immunoblotting with indicated antibodies. C) The indicated plasmids were transfected into HEK293T cells, and FLAG-TRAF6 was immunoprecipitated and used for *in vitro* ubiquitination reactions with ATP and recombinant purified Ub, UBE1, and UBC13-UEV1A. *In vitro* reactions were stopped after 15 minutes, and the E3 ubiquitin ligase activity of immunoprecipitated TRAF6 was revealed by immunoblotting for total ubiquitin as well as immunoblotting for anti-FLAG to detect total immunoprecipitated TRAF6. Total levels of ubiquitinated TRAF6 (Total UB, arbitrary units) were also quantified by densitometric scanning (IB: UB, all proteins products detected above ∼55–60 kDa). Data in (A–C) are representative of at least three independent experiments.

To better understand if PP1-γ activity influences the ubiquitination of TRAF6 as well as downstream targets, we first co-expressed TRAF6, IKKγ and PP1-γ and collected lysates for western blot. At lower exposures, we observed a single higher molecular weight band of IKKγ that was present when PP1-γ and IKKγ were co-expressed (Figure 5A; top panel, lane 4), and this band was consistent with ubiquitinated IKKγ detected in previously published studies [7], [39], [40]. The approximate sizes and banding patterns of larger protein species observed at longer exposures suggested that they might represent mono-, di-, or poly-ubiquitin conjugates of IKKγ. Importantly, the catalytic activity of PP1-γ was necessary for these observed IKKγ post-translational modifications (Figure 5A; top panel, lane 5). We also observed higher molecular weight banding of TRAF6 in anti-FLAG immunoblots at higher exposures (Figure 5A; middle panel, lane 4) associated specifically with the expression of catalytically active PP1-γ, further supporting a role for PP1-γ in the regulation of TRAF6 E3 ligase activity.

To confirm that the post-translational modification of IKKγ observed in Figure 5A did represent an ubiquitination event, we probed for direct ubiquitination of IKKγ by immunoprecipitation of IKKγ proteins that were lysed in an SDS buffer and boiled, followed by immunoblot to detect ubiquitin conjugates (Figure 5B). We observed that PP1-γ significantly increased the amount of ubiquitin conjugated to IKKγ. This PP1-γ-dependent increase was further enhanced after TLR7 stimulation (Figure 5B).

Based on our data that PP1-γ physically associates with both TRAF6 and IKKγ (Figure 4A), and influences IKKγ post-translational modifications (Figure 5A, B), we hypothesized that PP1-γ directly regulates the E3 ubiquitin ligase activity of TRAF6. To test whether PP1-γ catalytic activity regulates TRAF6 autoubiquitination, we co-expressed PP1-γ WT or D64N together with TRAF6, and then assayed the activity of immunoprecipitated TRAF6 through an *in vitro* autoubiquitination assay (Figure 5C). Using this approach, catalytically active PP1-γ enhanced the E3 auto-catalytic activity of TRAF6 more than 50% compared to vector-transfected conditions (Figure 5C, compare lanes 2 and 3), as measured by increased high molecular weight species in anti-ubiquitin (UB) immunoblots. In contrast, PP1-γ D64N repressed TRAF6 autoubiquitination (Figure 5C, compare lanes 2 and 4), consistent with our finding that catalytically inactive PP1-γ acts to inhibit NF-κB signaling. This data suggests that a PP1-γ-dependent dephosphorylation event is a critical prerequisite for TRAF6 E3 ubiquitin ligase activity.

### Macrophages Require PP1-γ for an Optimal Proinflammatory Response

**Figure 6:**
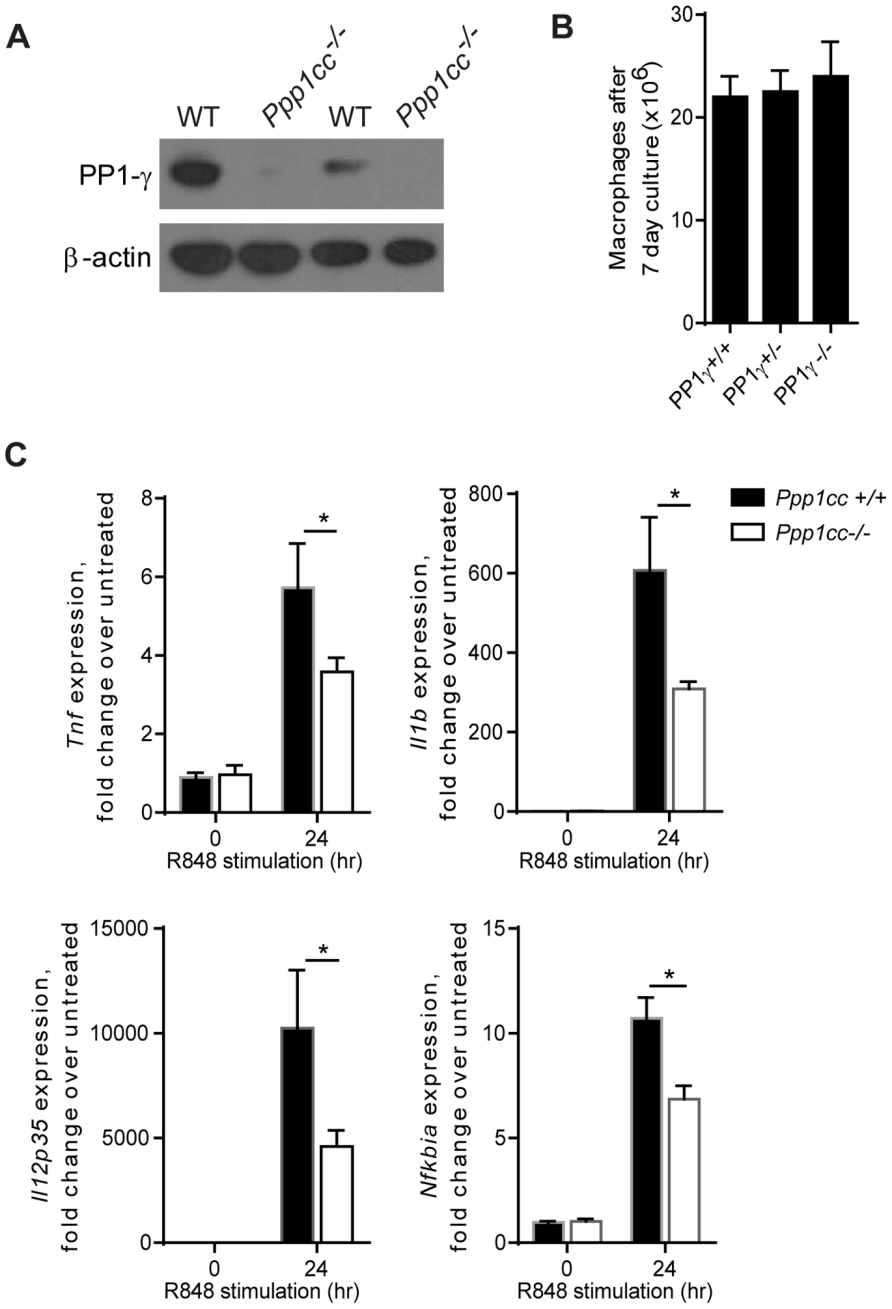
Macrophages require PP1-γ for an optimal proinflammatory response. A) Lysates were generated from *Ppp1cc^+/+^* or *Ppp1cc^−/−^* BMDM, and the level of PP1-γ in each lysate was evaluated by western blot. Lysates in lanes 1 and 2 are from one mouse each, and the lysates in lanes 3 and 4 are from two pooled mice each B) The number of BMDM was measured after 7 days of culture. The data shown are the average of at least 4 mice for each genotype. C) Expression of *Tnf, Il1b, Il12p35* and *Nfkbia* was measured after stimulation with R848 (10 µM) for the indicated amount of time. For (C), the graphs are an average of 5 *Ppp1cc^+/+^* and 7 *Ppp1cc^−/−^* mice. The data were pooled from two independent experiments and are shown as the mean ± SE; by unpaired, one-tailed student’s t test, *P*≤0.05 = *.

In order to study the role of PP1-γ in a more physiologically relevant cell type, we utilized bone-marrow derived macrophages (BMDM) from a *Ppp1cc^−/−^* mouse [28]. We first confirmed by western blot that PP1-γ was absent in BMDM from *Ppp1cc^−/−^* animals (Figure 6A). There was no difference in the number of macrophages that were derived from the wild type or *Ppp1cc^−/−^* bones, indicating that an absence of *Ppp1cc* does not affect macrophage development (Figure 6B). Wild type and *Ppp1cc^−/−^* BMDM were then stimulated with R848 for 24 hours, and the induction of proinflammatory gene transcripts was measured. There was a significant reduction in *Tnf*, *Il1b, Il12p35* and *Nfkbia* transcript levels in *Ppp1cc^−/−^* macrophages after 24 hr of R848 stimulation (Figure 6C). In contrast, induction of *Isg54* after TLR3 stimulation with polyI:C was similar in both *Ppp1cc^+/+^* and *Ppp1cc^−/−^* macrophages, further supporting a role for PP1-γ downstream of MyD88 (Supplementary Fig. S3). These data indicate that PP1-γ plays a critical role in sustaining a macrophage proinflammatory response.

### PP1-γ Regulates Inflammatory Responses during Pathogen Infection

**Figure 7:**
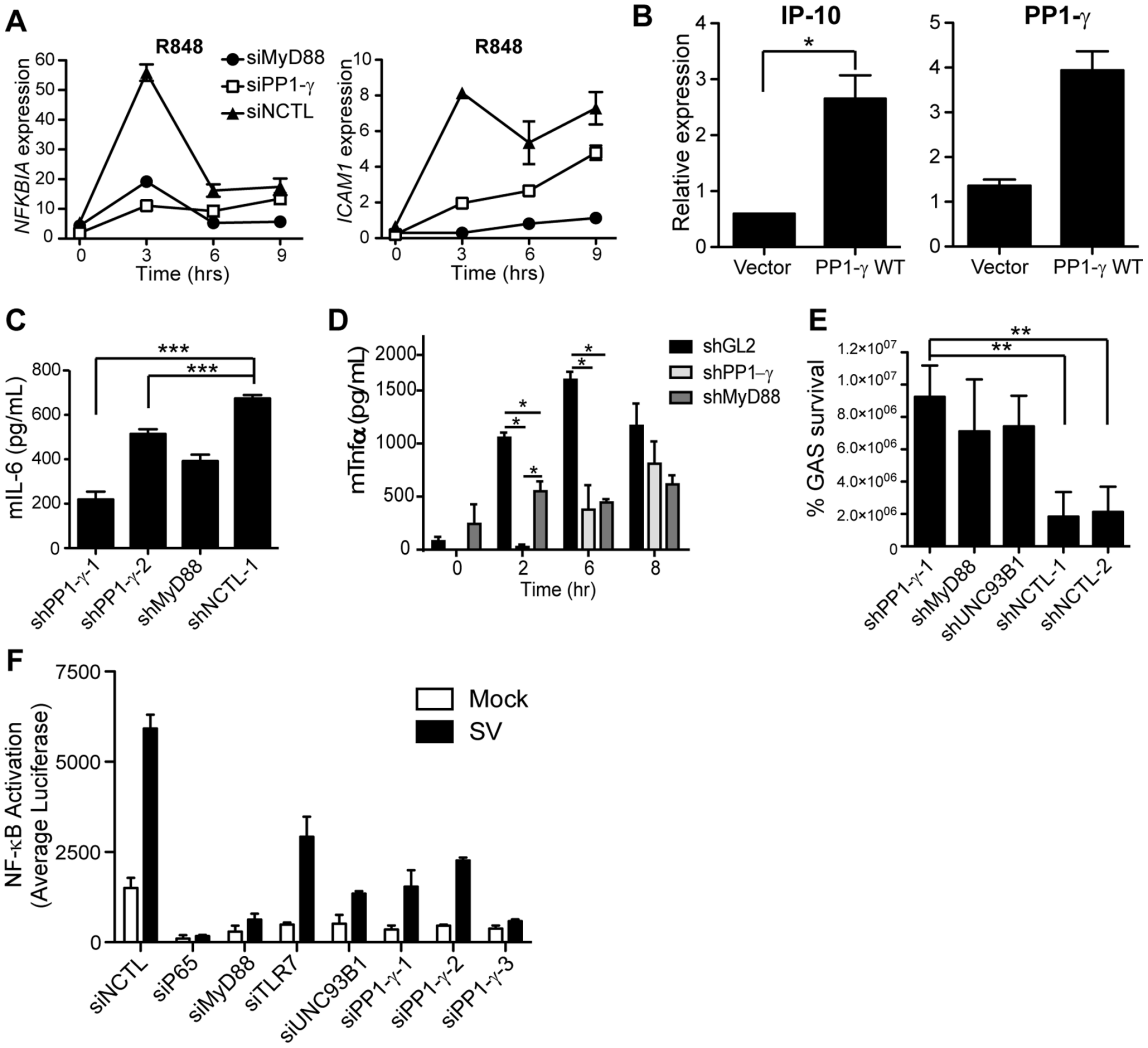
PP1-γ is a key component of innate immune responses in myeloid-lineage cells. A) A THP-1 monocytic cell line was transfected with the indicated siRNAs. Cells were stimulated with R848 (10 µM) for 0, 3, 6, and 9 hours, and relative levels of ICAM-1 and IκBα mRNA were evaluated by RT-PCR. Also see Figure S4A. B) Human primary monocyte-derived dendritic cells (MDDCs) were transduced with the lentiviral vectors harboring indicated cDNAs. Forty-eight hours later, samples were treated with LPS (100 ng/mL, 4 h). Total mRNA was purified, and relative levels of PP1-γ mRNA and LPS-induced IP-10 mRNA levels were quantified by RT-PCR. C) RAW264.7 cells were transduced with the indicated shRNAs and selected with puromycin to establish stable cell lines. Subsequently, knockdown cell lines were stimulated with LPS (100 ng/mL) for 12 hours, and secreted murine IL-6 was quantified by ELISA. D) RAW264.7 stable knockdown cell lines described in (C) were stimulated with R848 (10 µM) for indicated time and murine TNF was measured by ELISA. E) RAW264.7 stable knockdown cell lines described in (C) were infected with group A *Streptococcus* (GAS). Four hours later, total surviving bacteria were quantified by cell harvesting and lysis, followed by enumeration of bacterial colony forming units (cfu). F) HEK293T/TLR7/NF-κB-luc cells were transfected with the indicated siRNAs together with an siRNA targeting RIG-I. Subsequently, cells were infected with Sendai virus and luciferase reporter activity was quantified. Data shown in (A, and C–E) are representative of at least three independent experiments and are presented as mean ± SD from a representative experiment. For panel (D), *P*≤0.05 = *, as determined by one-way ANOVA with Tukey’s post-test. Data shown in (B) is representative sample of four out of 6 donors; by two-tailed student’s t test, *P*≤0.05 = *, *P*≤0.01 = **, *P*≤0.001 = ***.

We next examined the role of PP1-γ in innate immune responses of various myeloid cell types. The human monocytic cell line, THP-1, can respond to stimulation with multiple TLR ligands and represents an innate cell type responsible for pathogen recognition. In this cell line, RNAi against PP1-γ impaired the kinetics of R848-induced *ICAM1* and *NFKBIA* mRNA upregulation over a time course of receptor stimulation (Figure 7A and Supplementary Fig. S4A). Importantly, impaired induction of both I-CAM1 and IκBα was observed as early as three hours post-stimulation. Additionally, forced expression of PP1-γ WT in primary monocyte-derived dendritic cells (MDDCs) caused enhanced induction of the cytokine IP-10 following LPS stimulation (Figure 7B). Finally, stable silencing of PP1-γ in RAW264.7 mouse macrophages impaired secretion of IL-6 following LPS stimulation and TNF-α secretion following R848 stimulation (Figure 7C, D; Supplementary Fig. S4B). To determine the functional relevance of PP1-γ during the innate response to microbial infection, PP1-γ RAW264.7 knockdown cell lines were also infected with group A *Streptococcus* (GAS). Signaling through TLR9 has been implicated in the innate control of GAS [3] and our previous work has confirmed that intact endosomal TLR responses are critical for the control of this pathogen [41]. Importantly, when RAW264.7 macrophages with stable knockdown of PP1-γ were infected with GAS [27], silencing of PP1-γ resulted in diminished macrophage bactericidal activity, suggesting that the activity of this phosphatase in NF-κB signalling is critical for TLR9-dependent sensing and innate immune responses to GAS (Figure 7E). In addition to mediating innate detection of bacterial genomes, PP1-γ regulates inflammatory responses to Sendai virus (SV), a negative sense, single-stranded RNA paramyxovirus. As SV infection elicits a robust RIG-I-mediated immune response [1], we evaluated the effect of PP1-γ RNAi in cells where RIG-I was also silenced. In HEK293T cells that are deficient in RIG-I and infected with SV, silencing of PP1-γ by three independent siRNAs significantly attenuated the immune response to viral infection (Figure 7F). Notably, the reduction in SV-mediated NF-κB activation associated with PP1-γ RNAi was similar to that observed following knockdown of Unc93B1, a trafficking chaperone required for TLR7/9 endosomal delivery and signaling. Together, these data demonstrate that PP1-γ is a critical regulator of TLR-directed innate immunity, and is an important molecular component of the proinflammatory response to microbial infection.

## Discussion

Here, we demonstrate a role for PP1-γ in the positive regulation of TRAF6-mediated proinflammatory signaling and innate immune activation. Several complementary lines of genetic and biochemical evidence indicate a specific function for PP1-γ in Toll/IL-1R-dependent signaling pathways. Ligand profiling studies revealed that PP1-γ was exclusively required for transcriptional induction of NF-κB target genes downstream of TLR4/5/7 and IL-1R, but not downstream of TLR3 or TNFR (Figure 1G and Supplementary Fig. S1C). As the former set of receptors use MyD88 to initiate proinflammatory responses while TLR3 and TNFR do not, these findings support the conclusion that this phosphatase is necessary for signaling initiated via the MyD88 adaptor and is dispensable for signaling mediated by adaptors TRIF or TRADD/RIP. This observation was further supported by biochemical mapping studies (Figure 3A–C and Supplementary Fig. S2). Moreover, we find that PP1-γ physically interacts with signaling molecules associated with MyD88-dependent NF-κB responses such as TRAF6, IKKγ, and TAK1, but does not interact with proteins required for IRF-responses such as TBK1 or IKKε (Figure 4A). Based on the known circuitry of Toll/IL-1R and TNFR signaling cascades, these data support a model where PP1-γ regulates proinflammatory innate immune pathways initiated via a TLR-dependent MyD88-TRAF6 signaling axis.

A recent study by Wies and colleagues demonstrated a role for PP1-γ and PP1-α in the dephosphorylation of innate immune cytoplasmic RNA sensors RIG-I and MDA5 (RIG-I-like receptors, RLRs) resulting in downstream production of IFNβ [17]. Our findings further extend the role of PP1-γ in innate immunity by demonstrating that it is a critical regulator of MyD88-dependent TLR responses during microbial infection. One striking difference that exists between our findings and those of Wies and colleagues is that we demonstrate that PP1-γ associates with TRAF6 and IKKγ under basal conditions in the absence of infection. In contrast, PP1-γ and PP1-α were only found to associate with RIG-I and MDA5 after SeV treatment. This distinction indicates that PP1-γ plays a critical constitutive role in controlling TLR-mediated NF-κB activation, while the role and requirement of this phosphatase for RLR signaling may be under spatial or temporal regulation during receptor stimulation. Regarding PP1-α, a comparison of our findings with those of Weis et al. suggest that this subunit may be selectively required for IFN production downstream of RLRs, as we find that overexpression of the α subunit does not result in TLR-mediated innate signaling.

Although PP1-γ regulation of RLRs appears to be mechanistically distinct from its role in TLR signaling, it is unclear if coincident regulation of these pathways by PP1-γ reflects potential cross-talk between the cytoplasmic and membrane-associated pattern recognition receptor responses. Intriguingly, MAVS, a crucial adapter molecule for RIG-I, also has been demonstrated to interact with both TRAF6 and IKKγ to activate both NF-κB and IRF3 [42], [43]. Further studies will provide insight as to whether PP1-γ is able to directly interact with and regulate MAVS.

In addition to the regulation of RLRs and TLRs by PP1-α and PP1-γ, the catalytic activity of threonine phosphatase EYA4 also enhances antiviral responses to several viruses known to be recognized in a TLR-dependent manner [44]. This phenomenon wherein phosphatases act as activators of innate immunity is in contrast to the model that primarily kinases act as signaling components that promote proinflammatory pathway activation, such as the activation of IKKα/β by TAK1 [8]. Typically, phosphatases are implicated in resolving, dampening, or fine-tuning these responses by dephosphorylating enzymes to terminate downstream signaling and control inflammation [18], [45]. Our study highlights the emerging role of protein dephosphorylation in activation of PRR signaling, and further demonstrates the critical nature of these enzymes for successful host defense against harmful microorganisms.

Our data supports a direct role for the enzymatic activity of PP1-γ in TLR pathway activation, as the ubiquitin ligase activity of TRAF6 was diminished by the absence of PP1-γ phosphatase activity. These findings initially suggest that a TLR pathway component is constitutively phosphorylated by an unknown kinase to attenuate innate responses to sub-threshold stimuli. Subsequently, PP1-γ activity would be required to dephosphorylate this component to drive inflammatory signaling during microbial infection. Because PP1-α has been characterized as an inhibitor of TNFR-induced NF-κB signaling [18], and our results demonstrate that PP1-γ is an activator of MyD88-dependent inflammatory responses, there may be multiple phosphatases that act in concert to provide checkpoints for activation or termination of signaling in order to balance productive immune responses and chronic inflammation.

While our work does show a requirement of PP1-γ for activation of proinflammatory responses, several open questions remain. One in particular is how the phosphatase activity of PP1-γ is able to regulate an E3 ubiquitin ligase. It is possible that PP1-γ may specifically dephosphorylate an unknown inhibitory phospho-site on TRAF6, its E2 enzyme complex, or one of its substrates, allowing for full E3 ubiquitin ligase activity. Such a dephosphorylation event may expose nearby residues for modification by ubiquitin or may otherwise enhance the enzymatic activity of TRAF6, ensuring a kinetically robust response to pathogen challenge. Alternatively, the target of PP1-γ activity may be a deubiquitinating enzyme such as A20 or CYLD that is known to deactivate TRAF6 and IKKγ by removing ubiquitin conjugates [7], [46], [47]. Interestingly, the association of CYLD with IKKγ coincides with the appearance of a phosphorylated form of CYLD, though it has not been shown that this modification is required for interaction and deubiquitinating activities [7]. Due to the importance of both NF-κB and IFN signaling in the proper clearance of bacterial and viral infections, it will be of great interest to elucidate the exact mechanism governing the innate immune function of PP1-γ. Altogether, the data presented here defines the phosphatase PP1-γ as a positive regulator of MyD88-dependent TLR signaling and provides critical insight into the molecular events that regulate TRAF6 activity. Furthermore, our work emphasizes the expanding role of phosphatases in promoting innate responses to pathogen challenge.

## Supporting Information

Figure S1 **Silencing of PP1-γ impairs induction of MyD88-dependent proinflammatory cytokines.** A) HEK293T/NF-κB-luc cells stably expressing TLR7 (HEK293T/TLR7/NF-κB-luc) were reverse transfected with the indicated siRNAs. Forty-eight hours post-transfection, cells were stimulated for 16 h with R848 (10 µM), and NF-κB activation was measured by luciferase, and relative levels of PP1-γ or IL-8 were evaluated by RT-PCR. Also see Figure 1C. B) HEK293T/TLR7/NF-κB-luc cells were transfected with the indicated siRNAs and stimulated with R848 (3 µM) for 12 hours. Total cellular RNA was collected from each sample and used to measure relative levels of PP1-γ mRNA by RT-PCR. C) HEK293T/NF-κB cells stably expressing TLR3, TLR4, or TLR7 were reverse transfected with the indicated siRNAs. Forty-eight hours post-transfection, cells were stimulated for 3 h with LPS (TLR4, 100 ng/mL), Flagellin (TLR5, 100 ng/mL), R848 (TLR7, 10 µM), or poly I:C (TLR3; 50 ug/mL). For evaluation of TLR5 signaling, HEK293T/NF-µB cells stably expressing TLR7 were used. After ligand treatment, the relative levels of TNF-α mRNA were evaluated by RT-PCR. Also see Figure 1E. Data in (A–B) are representative of at least three independent experiments, data in (C) is representative of at least two independent experiments; by two-tailed student’s t test, *P*≤0.05 = *, *P*≤0.01 = **, N.S. = not significant. Bar graphs are presented as the mean relative mRNA levels ± SD from a representative experiment.
(TIF)

Figure S2 **PP1-γ silencing does not impair NF-κB signaling events downstream of TNFR.** HEK293T/TLR7/NF-κB-luc cells were reverse transfected with the indicated siRNAs. Seventy-two hours later, cells were stimulated with TNF-α (10 ng/mL) for the indicated timepoints, and whole cell lysates were collected and evaluated by SDS-PAGE and immunoblotting with the indicated antibodies. Data shown are representative of at least three independent experiments.
(TIF)

Figure S3 **TLR3 signaling is unchanged in** ***Ppp1cc*** **-deficent macrophages.** Expression of *Isg54* was measured after stimulation with polyI:C for the indicated amount of time. The graph shows the mean ± SEM of three mice for each genotype.
(TIF)

Figure S4 **Silencing of PP1-γ in THP-1 or RAW cell lines.** A) THP-1 monocytic cells were transfected with siRNAs. Cells were stimulated with R848 (10 µM) for the indicated time points, and relative levels of PP1-γ mRNA were evaluated by RT-PCR to confirm silencing of PP1-γ. Also see Figure 7A. B) RAW264.7 cells were transduced with the indicated shRNAs and stable cell lines were established as described. RNA was isolated from stable cell lines and relative levels of PP1-γ mRNA were evaluated by RT-PCR to confirm silencing of PP1-γ. Also see Figure 7C–E.
(TIF)

Table S1 **Candidate gene hits from the secondary confirmation screen are listed in descending order of NF-κB luciferase reporter activation.** The Z primes of the first and second runs of the screen were 0.72 and 0.62, respectively. The negative and positive controls in are in red.
(XLSX)
