# Supplementary figures and images for: Positive Regulation of TRAF6-Dependent Innate Immune Responses by Protein Phosphatase PP1-γ
Source: PLoS One. 2014 Feb 20;9(2):e89284. doi: 10.1371/journal.pone.0089284 (PMC3930702; doi:10.1371/journal.pone.0089284)

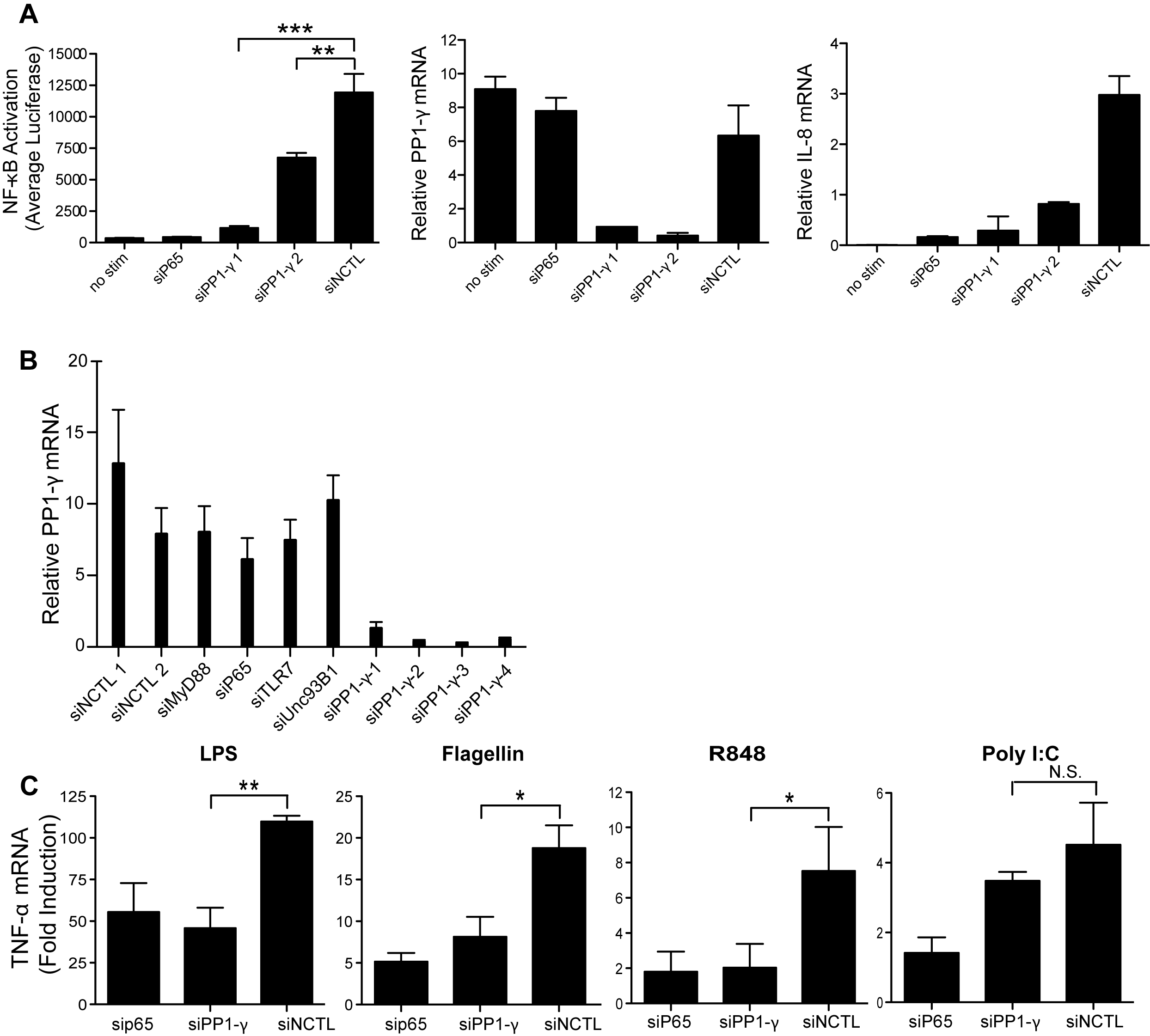

Supplement: Figure S1 — Silencing of PP1-γ impairs induction of MyD88-dependent proinflammatory cytokines. A) HEK293T/NF-κB-luc cells stably expressing TLR7 (HEK293T/TLR7/NF-κB-luc) were reverse transfected with the indicated siRNAs. Forty-eight hours post-transfection, cells were stimulated for 16 h with R848 (10 µM), and NF-κB activation was measured by luciferase, and relative levels of PP1-γ or IL-8 were evaluated by RT-PCR. Also see Figure 1C. B) HEK293T/TLR7/NF-κB-luc cells were transfected with the indicated siRNAs and stimulated with R848 (3 µM) for 12 hours. Total cellular RNA was collected from each sample and used to measure relative levels of PP1-γ mRNA by RT-PCR. C) HEK293T/NF-κB cells stably expressing TLR3, TLR4, or TLR7 were reverse transfected with the indicated siRNAs. Forty-eight hours post-transfection, cells were stimulated for 3 h with LPS (TLR4, 100 ng/mL), Flagellin (TLR5, 100 ng/mL), R848 (TLR7, 10 µM), or poly I:C (TLR3; 50 ug/mL). For evaluation of TLR5 signaling, HEK293T/NF-µB cells stably expressing TLR7 were used. After ligand treatment, the relative levels of TNF-α mRNA were evaluated by RT-PCR. Also see Figure 1E. Data in (A–B) are representative of at least three independent experiments, data in (C) is representative of at least two independent experiments; by two-tailed student’s t test, P≤0.05 = *, P≤0.01 = **, N.S. = not significant. Bar graphs are presented as the mean relative mRNA levels ± SD from a representative experiment. (TIF) [file pone.0089284.s001.tif]

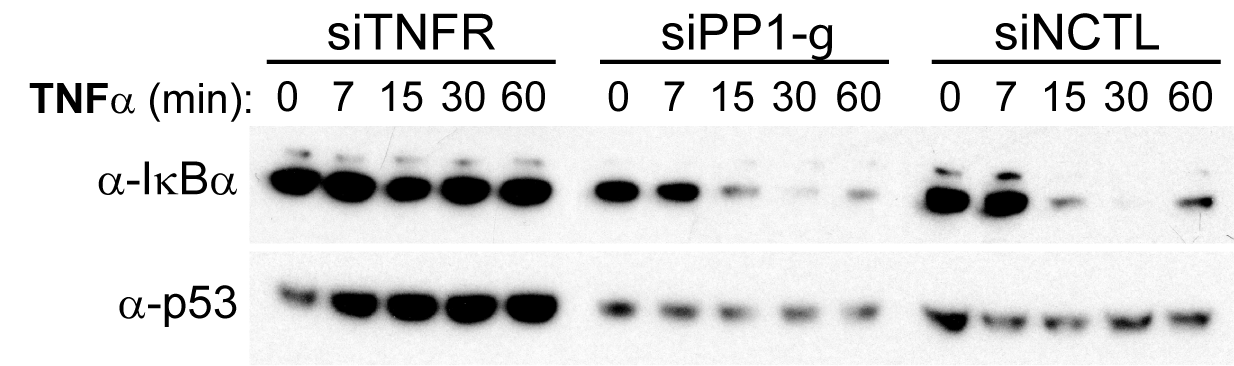

Supplement: Figure S2 — PP1-γ silencing does not impair NF-κB signaling events downstream of TNFR. HEK293T/TLR7/NF-κB-luc cells were reverse transfected with the indicated siRNAs. Seventy-two hours later, cells were stimulated with TNF-α (10 ng/mL) for the indicated timepoints, and whole cell lysates were collected and evaluated by SDS-PAGE and immunoblotting with the indicated antibodies. Data shown are representative of at least three independent experiments. (TIF) [file pone.0089284.s002.tif]

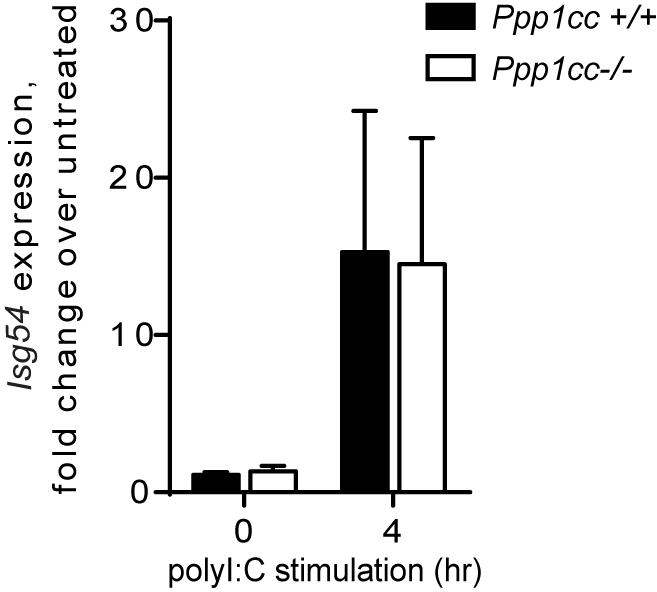

Supplement: Figure S3 — TLR3 signaling is unchanged in Ppp1cc -deficent macrophages. Expression of Isg54 was measured after stimulation with polyI:C for the indicated amount of time. The graph shows the mean ± SEM of three mice for each genotype. (TIF) [file pone.0089284.s003.tif]

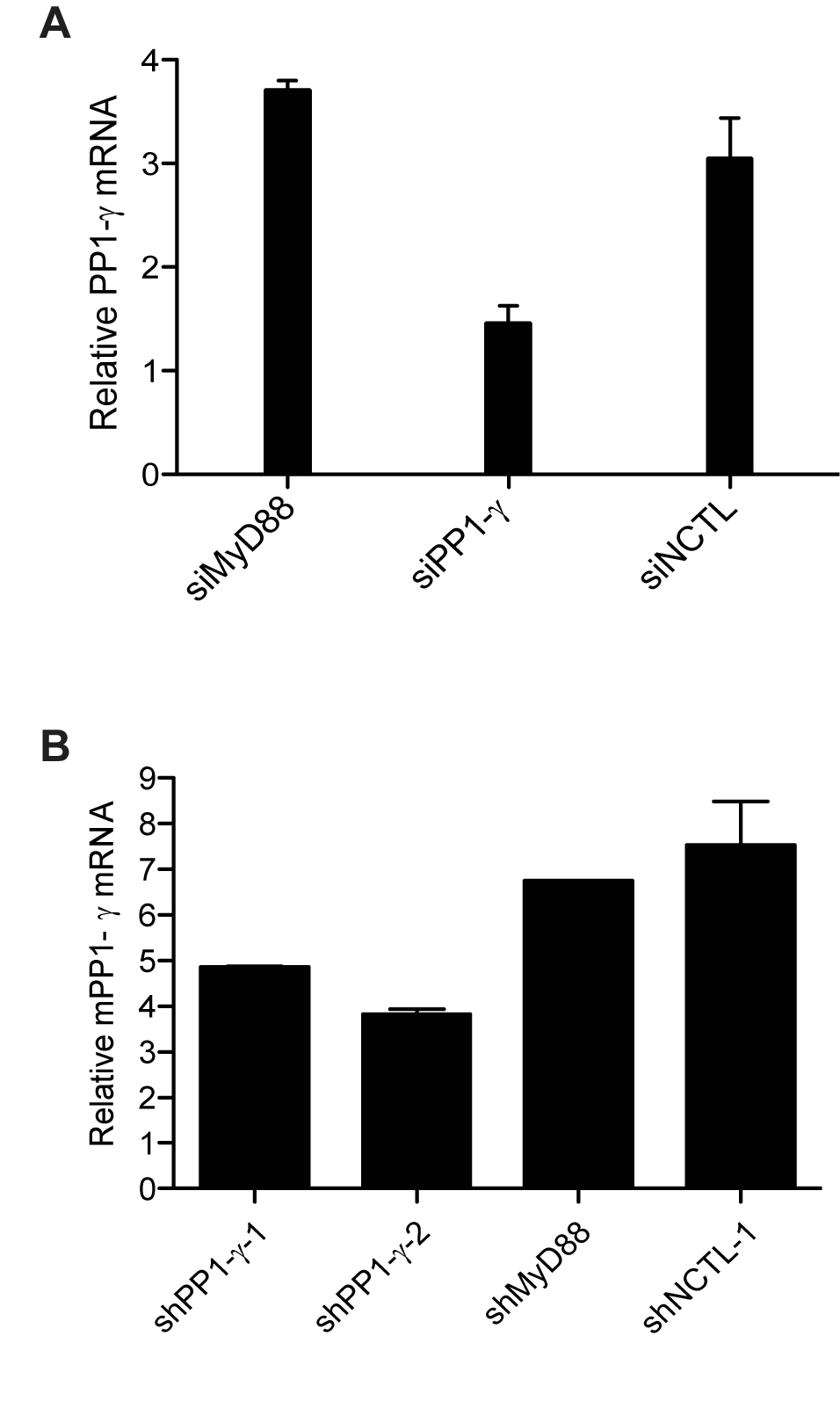

Supplement: Figure S4 — Silencing of PP1-γ in THP-1 or RAW cell lines. A) THP-1 monocytic cells were transfected with siRNAs. Cells were stimulated with R848 (10 µM) for the indicated time points, and relative levels of PP1-γ mRNA were evaluated by RT-PCR to confirm silencing of PP1-γ. Also see Figure 7A. B) RAW264.7 cells were transduced with the indicated shRNAs and stable cell lines were established as described. RNA was isolated from stable cell lines and relative levels of PP1-γ mRNA were evaluated by RT-PCR to confirm silencing of PP1-γ. Also see Figure 7C–E. (TIF) [file pone.0089284.s004.tif]
